# Influence of demographic change on the demand for radiotherapy using forecasted predictions for prostate cancer in Germany

**DOI:** 10.1007/s00066-023-02133-2

**Published:** 2023-08-28

**Authors:** M. Sonnhoff, M. Graff, K. Paal, J.-N. Becker, R.-M. Hermann, H. Christiansen, M. Nitsche, R. Merten

**Affiliations:** 1 Department of Radiotherapy and Special Oncology, Hannover Medical School, 30625 Hannover, Germany; 2 Center for Radiotherapy and Radiooncology Bremen and Westerstede, 28239 Bremen, Germany; 3 Depatment für Radiotherapy University Hospital Graz, 8036 Graz, Austria

**Keywords:** Projection, Prostat-cancer, Radiotherapy

## Abstract

**Purpose:**

Demographic change will lead to an increase in age-associated cancers. The demand for primary treatment, especially oncologic therapies, is difficult to predict. This work is an attempt to project the demand for radiation therapy (RT) in 2030, taking into account demographic changes using prostate cancer (PC) as an example.

**Materials and methods:**

Using the GENESIS database of the Federal Statistical Office, we retrieved demographic population projections for 2030 and retrospective demographic surveys from 1999 to 2019. Additionally, we queried incidence rates for PC in the respective age groups of 50–54, 55–59, 60–64, 65–69, 70–74, 75–79, 80–84, and +85 years from 1999–2019 via the Federal Cancer Registry of the Robert Koch Institute. We used a regression method to determine the age-dependent correlation between the incidence of PC and the population size of the respective age group by combining the data from 1999 to 2019. This information was used to calculate the incidence rates in the age groups of the expected population for 2030 and the expected new cases of PC in 2030. Finally, we extrapolated the indications for the demand for RT based on data from the Report on Cancer Incidence in Germany from 2016.

**Results:**

Considering a population-dependent incidence rate, an increase in new cases of PC is expected. This increase is particularly evident in the age groups of 70–74 and 80–84 years. With regards to RT, the estimate indicates an overall increase of 27.4% in demand. There is also a shift in RT demands towards older patients, especially in the 80- to 84-year-old age group.

**Conclusion:**

We observe an age-associated increase in primary cases of PC. This is likely to result in an increased demand for RT. The exact demand cannot be predicted. However, trends can be estimated to plan for the demand. This, though, requires a good database from cancer registries.

**Supplementary Information:**

The online version of this article (10.1007/s00066-023-02133-2) contains supplementary material, which is available to authorized users.

## Introduction

The predictions of the Federal Statistical Office foresee a substantial increase in the population proportion over 60 years of age in the context of demographic change [1]. At the same time, an increase in cancer cases is expected within this development [2]. This results in a rising demand for care and treatment. However, no estimates are currently available regarding the extent of the available resources.

The data of the German Centre for Cancer Registry Data provide excellent information on the incidence rates for most entities throughout Germany. However, more data on different oncologic therapeutic demands radiation therapy (RT), surgical treatment, and systemic therapies in Germany are urgently needed.

Only historical collectives with limited significance for current and future events are available. Information on the required therapies in connection with current trends can help estimate future needs and ensure maintenance of sufficient resources [3]. It is essential to see the demographic change in the German society as a potential driver for a rising rather than a declining demand [4].

Estimates are available for the most common entities in the clinical field [2], but also for rare entities such as thyroid cancer [5]. Some studies deal with the demand for chemotherapy [6]. Still, to our knowledge, no estimates are currently available regarding the demand for RT. Prostate cancer (PC) is among the most common entities with a high societal and individual disease burden [7, 8]. In addition, valid estimates of the expected primary cases in the future are already available, but data on demand for RT are still lacking.

How does demographic change influence the incidence of PC cases, and how does this influence the demand for RT?

## Methods

This work estimates the demographic influence on the incidence of PC in Germany. In addition, we estimate the impact of these changing incidences on the requirements for RT.

We captured the incidence values for PC from 1999 to 2019 for the age cohorts 50–54, 55–59, 60–64, 65–69, 70–74, 75–79, 80–84, and +85 years from the German Centre for Cancer Registry Data of the Robert Koch Institute [9]. In addition, we retrieved the demographic survey from 1999 to 2019 and the population projection for 2030 for the respective cohorts via the GENESIS database of the Federal Statistical Office [1]. In the estimate, we assumed a substantial increase in life expectancy. The birth rate and the migration index are negligible because they have little or no influence on the age cohort relevant for RT. Based on the retrospective incidence and population data from 1999 to 2019, we used linear regression to determine the correlation between incidence rate and population size for each age cohort (Eq. 1).

Equation 1: *a* is defined as the regression line that describes the relationship between population size and the magnitude of the increase in incidence rate. The value *b* in the linear regression formula describes the baseline incidence rate of the specific age cohort, and *x *represents the population size for the corresponding age cohort to determine the incidence rate for 2030.

1 $$a = \frac{\left( N \times \sum (\text{Incidence} \times \text{Population}) - \sum (\text{Incidence}) \times \sum (\text{Population}) \right)}{(N \times \sum (\text{Incidence}^{2}) - (\sum \text{Incidence})^{2})N}$$

$$b=\sum \left(\text{iPopulation}\right)-a\times \frac{\sum \left(\text{Population}\right)}{N}$$

$$\text{estimated}\,\text{Incidence}\,for2030=ax+b$$

Using the 2030 population projections, we estimated the 2030 incidence value for the defined age cohorts. We calculated the expected cases of disease based on the estimated incidence and, assuming the RT demand of 2016 published in the cancer report 2016 [10], calculated the RT demand for 2030. Finally, we compared our estimation with the historical collective of 2016.

## Results

The calculated estimates of incidence values for 2030 in the age cohorts show an increase in most of the age cohorts, with the most pronounced increases in the groups 80–84 and +85 years. However, we also describe a decrease in the age groups 60–64, 70–74, and 75–79 years (Table 1).

**Table 1:** Comparison of incidences recorded in 2016 with incidences estimated for 2030

Age cohort (years)	Incidence per 100,00 in 2016 [10]	Estimated incidence per 100,00 in 2030
50–54	52.3	97.6
55–59	144.6	167.9
60–64	299.7	278.8
65–69	506.6	391.2
70–74	656.9	555.2
75–79	679.2	794.4
80–84	584.8	1084.9
+85	610.4	1698.6

The estimate of the absolute number of cases shows no increase in the number of new cases in the 60–64 and 65–69 years cohorts, despite the falling incidence value of the estimate for 2030. We expect an increase in the absolute number of cases and, specifically, in the demand for RT in all cohorts except for the age cohorts 55–59 and 70–74 years. However, in both cases, this decrease is less than 10%. The absolute strongest increase is calculated for the group 80–84 years, while the relative percentage increase is estimated to be strongest in the +85 years cohort (Table 2). Overall, cohorts, new cases, and the need for radiotherapy are increasing.

**Table 2:** Comparison of new patients and RT demand from 2016 to 2030. We estimate the largest increase in the age cohorts 75–79 and 80–84 years, and in the group +85, which also shows the largest percentage increase

Age cohort (years)	New total cases in Germany 2016 [10]	New total cases in Germany 2030	Demand RT 2016 [10]	Estimated demand RT 2030	Change in demand RT	Change in demand RT (%)
50–54	1964	2459	361	452	+91	25.2
55–59	4362	4109	1001	943	−58	−5.8
60–64	7781	8511	2203	2410	+207	9.4
65–69	9778	11,918	3297	4019	+722	21.9
70–74	14,727	13,348	6103	5531	−572	−9.4
75–79	10,729	14,216	5234	6935	+1701	32.5
80–84	5445	13,307	1317	3219	+1902	144.4
+85	4143	19,991	383	1848	+1465	382.5
All	58,929	111,912	19,899	25,356	+5457	+27.4

*RT* radiation therapy

## Discussion

Within the estimate for 2030, we can describe an increase in incidences in almost all age cohorts. Moreover, the increases are significantly more significant than those in the just two age groups with decreases. This also influences the number of cases and, therefore, we expect an increase in the number of primary disease cases in 2030.

If the indications for RT in the primary situation were to be set according to the baseline situation of the historical collective of 2016, an increase in demand for RT in the primary disease case would also be expected here.

**Fig. 1:**
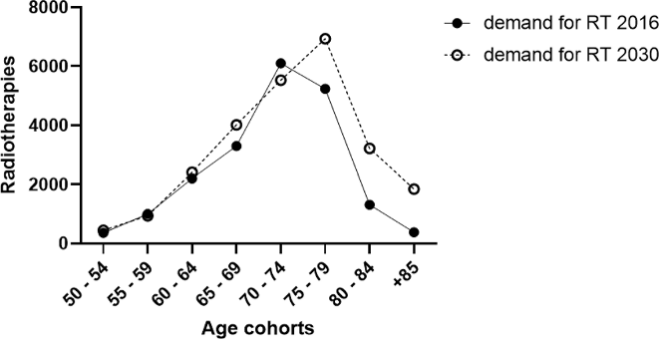
Comparison of radiotherapy demand of the historical collective with the estimated demand for 2030. (The increase is particularly noticeable in the age cohort 70–74 years) *RT* radiation therapy

The peculiarity here, however, is that there would be a shift to the right of the curve, and the largest proportion of absolutes would be expected in the 75–79 years age group (Fig. 1) simultaneously. There is only a slight drop in indications in the 60–64 and 65–69 years age groups. In both cases, treatment cases were reduced to below 10%.

Our estimates of total primary cases are comparable to a 2016 paper by Quante et al. The paper predicts a number of 121,335 primary cases of PC in 2030 [2]. However, our estimates are slightly lower, by about 10,000 cases.

Still, within the scope of our estimate, two points have influenced the number of cases. First, we neglect primary cases that occur at an age of younger than 50 years entirely in our estimation. In addition, we consider the excess mortality of the corona pandemic through the current population projection query [11]. Forecasting must adapt dynamically to changes and trends to provide an accurate projection.

A trend-adapted adjustment may also become relevant for further design of the screening strategy in Germany. The expansion of PSA screening affects the incidence of newly diagnosed PC [12]. Starting at the age of 45 years, statutory health insurance funds cover the costs of PSA screening. However, there is no screening call for the relevant age groups, as is the case, for example, with mammography for breast cancer. Recently published data showed the relation between the decreasing risk of cancer-specific death and early diagnoses in younger ages [13]. Therefore, expansion and adaptation of the screening strategy could also lead to a further increase in the number of cases and, thus, further raise the incidence rates in the cohorts.

However, in addition to the screening strategy, there are also difficulties in defining RT strategies in the context of the initial diagnosis. In the context of the query on the therapeutic approach in Germany, the German Centre for Cancer Registry Data was unable to provide us with any more current calculation basis beyond the historical collective of 2016 [10, 14]. Nevertheless, the indication for prostate RT in the primary situation is increasingly expanding, e.g., by including oligometastatic primary situations in the German healthcare reality with a wide range of recommendations from the German Society for Radiotherapy (DEGRO) [15]. This approach is supported by solid evidence from the Stampede study [16]. Furthermore, early initiation of therapy in the patient population of localized primary situations improved disease-free survival and reduced the need for antihormonal therapies [17].

Also, the decision on initial primary therapy needs to be further discussed. The UK ProtecT trial provides long-term data showing that RT is noninferior to surgical therapy for localized primary prostate cancer [17]. A more balanced approach to counseling could increase the trend towards RT in older age groups. The rightward shift of the curves compared to the historical collective shows the trend to irradiate older patients.

The tendency to treat older patients with RT and younger patients with surgery was a trend already evident in a retrospective analysis of treatment data from the Swedish healthcare system between from 1998 and 2012. While in the age cohort 60–64 years, the indication for surgery decreases, in the 70–74 years cohort, only 11% of patients receive surgery, and the indication for RT increases from 60–64 years of age (19.3%). In the 70–74 age cohort, the proportion is still 17.5% [18].

German data on the indication for primary prostatectomy in the same population would be very informative but are only available to a limited extent and insufficient to construct an accurate model. This information could be helpful to plan for care needs more accurately in the healthcare system.

In addition to the planning of healthcare delivery strategies, the late effects associated with treatment also play an essential role in the delivery of healthcare. Modern RT techniques increasingly reduce late complications and create an excellent posttherapeutic quality of life [19, 20]. However, a sufficient follow-up after the treatment remains indispensable for validation of the outcome. Not only must a sufficient number of specialists be available for application of the treatment itself, but structured aftercare must also be available.

A 2019 paper estimated the global demand for chemotherapy considering demographic change. The authors see an increasing demand of up to 51% more. In addition, an estimate was also given that this will also result in a corresponding need for qualified specialists [6]. Corresponding analogies can be derived for RT, indicating a rising demand for qualified specialists to meet the demand for treatment.

The data from the historical collective on the indication of 2016 also do not reflect the type and technique of RT or the indication (curative versus palliative). The collection of data consisted of indications for radiotherapy following initial diagnosis of legally insured persons in Germany [10]. However, it was not recorded whether the radiotherapy was indicated in the primary or metastatic situation. Therefore, a portion of the population may have also been treated with radiotherapy in the palliative setting, which would explain the constant proportion of patients over 80 years of age with indications for initial radiotherapy. Adequate representation of indications can only be achieved through better data transmission to cancer registries.

Furthermore, in addition to the indication, the type of administration of radiotherapy is also not represented. Besides conventional teletherapy, prostate brachytherapy is a very suitable treatment technique within its indication limits in terms of efficacy and toxicity [21]. However, this application is not available everywhere. Furthermore, hypofractionated techniques can also reduce the use of treatment facilities, but this approach does little to change the continuing need for specialists in treatment application, and it also requires adequate technical equipment, such as image guidance systems, to ensure the safety of treatment application [22]. Ultimately, it must also be considered that there is currently no clear German guideline recommendation for hypofractionation of prostate RT [23].

The demand for future treatment is never exactly estimable, but trends in population development and treatment prescriptions can help make estimates to plan resource-oriented care. Therefore, comprehensive reporting to the German cancer registries is essential to create this data basis.

### Supplementary Information

More detailed tables and graphics regarding the extrapolations presented here, as well as demographic descriptions, can be found in the supplement

## References

[1] (Destatis), D.S.B. (2023) Bevölkerungsvorausberechnung für 2030. Statistisches Bundesamt (Destatis), 2023, Accessed 20.03.2023

[2] Quante AS et al (2016) Projections of cancer incidence and cancer-related deaths in Germany by 2020 and 2030. Cancer Med 5(9):2649–2656 27356493 10.1002/cam4.767 PMC5055190

[3] Fietkau R et al (2023in) Strukturelle, prozedurale und personelle Voraussetzung für die Erbringung radioonkologischer und strahlentherapeutischer Leistungen 2023 in Deutschland – ein Positionspapier der Deutschen Gesellschaft für Radioonkologie (DEGRO). Strahlenther Onkol 199(8):697–705 37336797 10.1007/s00066-023-02105-6 PMC10361887

[4] Hermann S, Kraywinkel K (2019) Epidemiologie des Prostatakarzinoms in Deutschland. Onkologe 25(4):294–303 10.1007/s00761-019-0545-x

[5] Rahib L et al (2014) Projecting cancer incidence and deaths to 2030: the unexpected burden of thyroid, liver, and pancreas cancers in the United States. Cancer Res 74(11):2913–2921 24840647 10.1158/0008-5472.CAN-14-0155

[6] Wilson BE et al (2019) Estimates of global chemotherapy demands and corresponding physician workforce requirements for 2018 and 2040: a population-based study. Lancet Oncol 20(6):769–780 31078462 10.1016/S1470-2045(19)30163-9

[7] Winter A et al (2015) Increase in uro-oncological health care needs due to demographic change: extrapolation of cancer incidence numbers through 2030 as a basis for directed regional planning. Urologe A 54(9):1261–1268 25490922 10.1007/s00120-014-3698-7

[8] Porst M et al (2022) The burden of disease in Germany at the national and regional level. Dtsch Ärztebl Int 119(46):785–792 36350160 10.3238/arztebl.m2022.0314 PMC9902892

[9] Koch-Institut, Z.f.K.i.R. Datenbankabfrage mit Schätzung der Inzidenz, Prävalenz und des Überlebens von Krebs in Deutschland auf Basis der epidemiologischen Landeskrebsregisterdaten [Inzidenz, Prävalenz.. Accessed 13 Sept 2022

[10] Barnes B et al (2016) Bericht zum Krebsgeschehen in Deutschland 2016. Robert Koch-Institut

[11] Msemburi W et al (2023) The WHO estimates of excess mortality associated with the COVID-19 pandemic. Nature 613(7942):130–137 36517599 10.1038/s41586-022-05522-2 PMC9812776

[12] Welch HG, Albertsen PC (2020) Reconsidering prostate cancer mortality—The future of PSA screening. N Engl J Med 382(16):1557–1563 32294352 10.1056/NEJMms1914228

[13] Carlsson SV et al (2023) Young age on starting prostate-specific antigen testing is associated with a greater reduction in prostate cancer mortality: 24-year follow-up of the Göteborg randomized population-based prostate cancer screening trial. Eur Urol 83(2):103–109 36334968 10.1016/j.eururo.2022.10.006 PMC10481420

[14] Hager B et al (2015) Integrated prostate cancer centers might cause an overutilization of radiotherapy for low-risk prostate cancer: a comparison of treatment trends in the United States and Germany from 2004 to 2011. Radiother Oncol 115(1):90–95 25770874 10.1016/j.radonc.2015.02.024

[15] Müller AC et al (2022) Radiotherapy for hormone-sensitive prostate cancer with synchronous low burden of distant metastases. Strahlenther Onkol 198(8):683–689 35704054 10.1007/s00066-022-01961-y PMC9300516

[16] Parker CC et al (2018) Radiotherapy to the primary tumour for newly diagnosed, metastatic prostate cancer (STAMPEDE): a randomised controlled phase 3 trial. Lancet 392(10162):2353–2366 30355464 10.1016/S0140-6736(18)32486-3 PMC6269599

[17] Hamdy FC et al (2023) Fifteen-year outcomes after monitoring, surgery, or radiotherapy for prostate cancer. N Engl J Med, 2023 Apr 27;388(17):1547–1558. 10.1056/NEJMoa2214122 10.1056/NEJMoa2214122 36912538

[18] Pettersson A et al (2018) Age at diagnosis and prostate cancer treatment and prognosis: a population-based cohort study. Ann Oncol 29(2):377–385 29161337 10.1093/annonc/mdx742

[19] Lardas M et al (2017) Quality of life outcomes after primary treatment for clinically Localised prostate cancer: a systematic review. Eur Urol 72(6):869–885 28757301 10.1016/j.eururo.2017.06.035

[20] Hoeller U et al (2021) Late sequelae of radiotherapy—The effect of technical and conceptual innovations in radiation oncology. Dtsch Ärztebl Int 118(12):205–211 34024324 10.3238/arztebl.m2021.0024 PMC8278127

[21] Strouthos I et al (2022) High-dose-rate brachytherapy for prostate cancer: rationale, current applications, and clinical outcome. Cancer Rep 5(1):e1450 10.1002/cnr2.1450 PMC8789612 34164950

[22] Jereczek-Fossa BA et al (2019) Late toxicity of image-guided hypofractionated radiotherapy for prostate: non-randomized comparison with conventional fractionation. Radiol Med 124(1):65–78 30219945 10.1007/s11547-018-0937-9

[23] Leitlinienprogramm Onkologie (2021) S3-Leitlinie Prostatakarzinom, Langversion 6.2 (AWMF Registernummer: 043/022OL)

