# Supplementary material for: Influence of demographic change on the demand for radiotherapy using forecasted predictions for prostate cancer in Germany
Source: Strahlenther Onkol. 2023 Aug 28;200(8):671–5. doi: 10.1007/s00066-023-02133-2 (PMC11272801; doi:10.1007/s00066-023-02133-2)
Supplement: Supplementary file 1 — More detailed tables and graphics regarding the extrapolations presented here, as well as demographic descriptions, can be found in the supplement [file 66_2023_2133_MOESM1_ESM.docx]

Table 1:Overview table of reported data for 2016 related to new cases in 2016, radiotherapy needs identified from GKV data in 2016, alongside population projections for 2030, estimated incidence rates, and estimated radiation therapy needs. GKV (Statutory Healt)

| Agecohort [a] | New cases 2016 [n]  [1] | GKV with Radiotherapy 2016 [n] [1] | Radiotherapy 216 in percent [%] | Population 2030 [n]  [2] | Estimation of the Incidence 2030 [n] | | Estimated Need for Radiotherapy 2030 [n] |
| --- | --- | --- | --- | --- | --- | --- | --- |
| 50 - 54 | 1,964 | 361 | 18.4 | 2,519,700 | 97.6 | 2,459 | 452 |
| 55 - 59 | 4,362 | 1,001 | 22.9 | 2,447,500 | 167.9 | 4,109 | 943 |
| 60 - 64 | 7,781 | 2,203 | 28.3 | 3,052,700 | 278.8 | 8,511 | 2,410 |
| 65 - 69 | 9,778 | 3,297 | 33.7 | 3,046,600 | 391.2 | 11,918 | 4,019 |
| 70 - 74 | 14,727 | 6,103 | 41.4 | 2,404,100 | 555.2 | 13,348 | 5,531 |
| 75 - 79 | 10,729 | 5,234 | 48.8 | 1,789,500 | 794.4 | 14,216 | 6,935 |
| 80 - 84 | 5,445 | 1,317 | 24.2 | 1,226,600 | 1084.9 | 13,307 | 3,219 |
| 85 | 4,143 | 383 | 9.2 | 1,176,900 | 1698.6 | 19,991 | 1,848 |
| Summe | 58,929 | 19,899 | 33.8 | 17,663,600 | 633.55 | 111,912 | 37,790 |


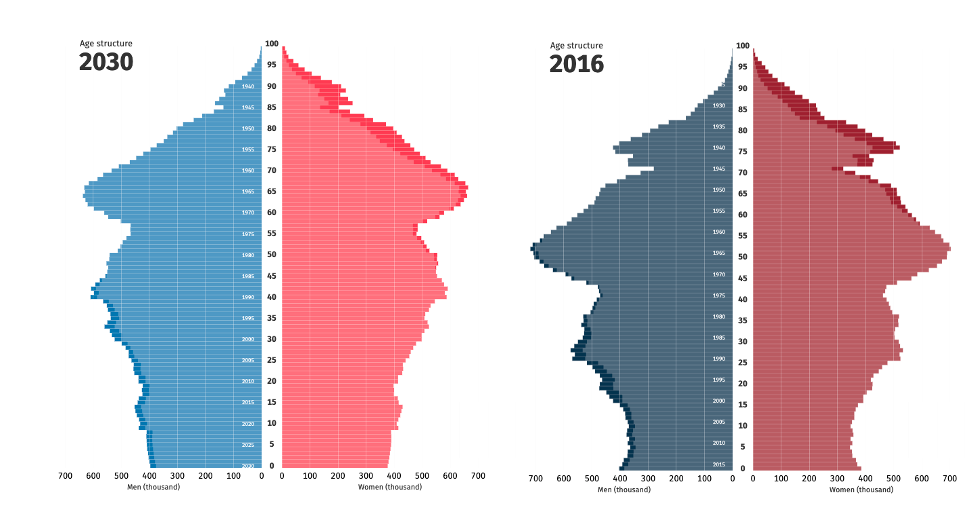


Fig. 1: Comparison of the age structure of the recorded population in 2016 to the projected population for 2030. © [Statistisches Bundesamt](https://www.destatis.de/impressum) (Destatis), Wiesbaden 2023 Access: 27.03.2033 [3]

Fig. 2: Diagram Comparing Cases Reported in 2016 with Our Estimation for 2030"

This diagram shows the number of cases reported in 2016 and our estimated number for 2030. The data is based on reliable sources and projections. In the diagram, the vertical axis represents the number of cases, and the horizontal axis shows the years. The blue line represents the cases reported in 2016, and the red line represents our estimation for 2030.

Fig. 3: Presentation of the incidence rate trend for the elderly over the period from 1999 to 2019. The division of the cohorts into two graphs was done for better clarity and overview.

Literature

1. Barnes B, Kraywinkel K, Nowossadeck E, Schönfeld I, Starker A, Wienecke A, Wolf U: **Bericht zum Krebsgeschehen in Deutschland 2016**. In*.*: Robert Koch-Institut; 2016.

2. (Destatis) DSB: **Bevölkerungsvorausberechnung für 2030**.

3. **14. koordinierte Bevölkerungsvorausberechnung für Deutschland** [<https://service.destatis.de/bevoelkerungspyramide/index.html#!y=2029>]
